# Relationship between uric acid and blood pressure in different age groups

**DOI:** 10.1186/s40885-015-0022-9

**Published:** 2015-07-15

**Authors:** Jae Joong Lee, Jeonghoon Ahn, Jinseub Hwang, Seong Woo Han, Kwang No Lee, Ji Bak Kim, Sunki Lee, Jin Oh Na, Hong Euy Lim, Jin Won Kim, Seung-Woon Rha, Chang Gyu Park, Hong Seog Seo, Dong Joo Oh, Eung Ju Kim

**Affiliations:** Cardiovascular Center, Korea University Guro Hospital, 148 Gurodong-ro, Guro-gu, 152-703 Seoul, South Korea; National Evidence-based Healthcare Collaborating Agency, Seoul, South Korea; Department of Cardiology, Dongtan Sacred Heart Hospital, Hallym University Medical Center, Hwaseong, South Korea

**Keywords:** Uric acid, Hyperuricemia, Blood pressure, Hypertension

## Abstract

**Introduction:**

Serum uric acid (UA) has been known to have a positive association with blood pressure (BP). However, the relationship between serum UA and BP in different age groups is unclear.

**Methods:**

A total of 45,098 Koreans who underwent health examinations at Korea Association of Health Promotion with no history of taking drugs related with UA and/or BP were analyzed for determining the relationship between serum UA and BP.

**Results:**

In men <40, serum UA was significantly associated with systolic (*β* = 0.25, *p* = 0.002) and diastolic BP (*β* = 0.41, *p* < 0.001) after adjustment for age, diabetes, dyslipidemia, body mass index, and estimated glomerular filtration rate. Men between ages 40 and 59 showed similar results regarding diastolic BP. The association between serum UA and BP was stronger in women <40 (*β* = 0.54, *p* < 0.001 for systolic BP; *β* = 0.65, *p* < 0.001 for diastolic BP) and in between 40 and 59 (*β* = 0.51, *p* < 0.001 for diastolic BP). The association was not significant in men and women ≥60. The odds ratios (ORs) of hyperuricemia for hypertension were 1.25 (95% confidence interval [CI], 1.08 to 1.45; *p* = 0.003) and 1.33 (95% CI, 1.11 to 1.60; *p* = 0.002) in men <40 and in between 40 and 59, respectively, in the multivariate analysis. The OR was 2.60 (95% CI, 1.37 to 4.94; *p* = 0.0034) in women <40. The relationship between hyperuricemia and hypertension was not significant in other age/gender groups.

**Discussion:**

In contrast to the elderly of 60 and over, the non-elderly showed significant associations between serum UA and BP.

## Introduction

Hypertension in adults is the most common form of cardiovascular diseases. The prevalence of hypertension grows higher with aging, resulting in an increase in morbidity and mortality through various events such as myocardial infarction, heart failure, stroke, and renal failure [1-4].

Hyperuricemia has been proposed to have an association with hypertension in various studies. Serum uric acid (UA) levels were demonstrated to be an independent predictor for developing hypertension [5-7]. Regardless of the different ethnic origins, a continuous relationship between serum UA and blood pressure (BP) was observed in African-Americans and whites [8,9] as well as in Asians [7,10] including Koreans [11-13]. For determining the causal role of serum UA in the development of hypertension, Mazzali et al. [14] demonstrated an elevation in serum UA followed by an increase in BP via a crystal-independent mechanism in rat models. Reduction of serum UA was associated with a decrease in BP through the regulation of renin-angiotensin and nitric oxide system [15].

Taking this into account, a hypothesis regarding the effect of serum UA-lowering agents which could have potential benefits in prevention and treatment of hypertension has emerged. Feig and Johnson [1] showed a high correlation between serum UA and BP in childhood primary hypertension and demonstrated promising results using allopurinol, a UA-lowering agent, to reduce BP in adolescents with newly developed hypertension in a pilot study [16]. Early intervention of controlling serum UA was proposed to have effect on delaying the progression of early hypertension. However, studies specifically of elderly patients have had controversial results regarding the relation between serum UA and BP [17,18]. Also, the use of UA-lowering agents did not have effect on controlling BP in the elderly as in the adolescents [19]. Taken together, the relationship between serum UA and BP was known to be weakened by the aging process, but there were no studies confirming in which age group the relationship would be the strongest.

Also, studies mentioned above lack control over variables which could have effect on serum UA levels such as the history of taking diuretics, antihypertensive medication or other drugs, obesity, and renal function. Moreover, some of them were performed with only a small number of subjects.

On the basis of the considerations above, we performed the present study to compare the relationship between serum UA and BP or hypertension by different age groups in a single large cohort with adjustment of all possible confounding factors. This study may identify a more specific target group in which the association between serum UA and BP or hypertension is the highest and controlling serum UA would have maximal benefit.

## Methods

### Subjects

A total of 91,882 subjects who underwent health examinations at Korea Association of Health Promotion (KAHP) during January, 2005 to December, 2009 were initially enrolled. Patients lacking at least 6 months of medical history in the Health Insurance Review and Assessment Service (HIRA) system were excluded. Informed consent was obtained according to the Helsinki Declaration for all of the enrolled patients in our study, and the study protocol was approved by the institutional review boards of KAHP, HIRA, and Korea University.

**Figure 1:**
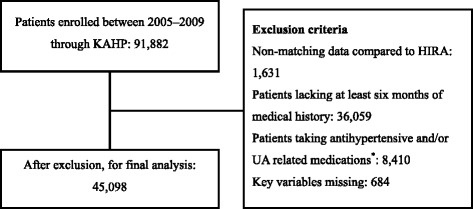
Flow diagram of the study protocol. *Non-antihypertensive medications which could affect serum UA levels were excluded including allopurinol, benzbromarone, colchicine, febuxostat, rasburicase, probenecid, diuretics, ethambutol, cyclosporine, diazoxide, aspirin, levodopa, and nicotinic acid. KAHP, Korea Association of Health Promotion; HIRA, Health Insurance Review and Assessment Service; UA, uric acid.

Subjects with a history of taking antihypertensive and/or medications which could affect serum UA levels were also excluded. A flow diagram of the study protocol and medications for exclusion are presented in Figure 1. A total of 45,098 subjects were included in the final analysis. To evaluate the relationship between serum UA and BP in different ages, we divided the subjects into three groups by age; <40, 40–59, and ≥60. In each group, comparison between gender differences was done also.

### Baseline measurements and definitions

Systolic and diastolic BP were measured in all of the subjects. The diagnosis of hypertension was based on a systolic BP of 140 mmHg or higher and a diastolic BP of 90 mmHg or higher. Height and weight were measured and body mass index (BMI) was calculated as the weight in kilograms (kg) divided by the square of the height in meters (m). Previous diagnoses which could affect serum UA levels, such as diabetes, chronic kidney disease, dyslipidemia, were thoroughly investigated. Fasting blood sugar levels, estimated glomerular filtration rate (eGFR), and lipid profiles including total cholesterol, triglyceride, high-density lipoprotein, and low density lipoprotein were checked for the evaluation of comorbidities. History of hospital admissions and previous medications were also taken through a cohort established between the network of KAHP and HIRA. Hyperuricemia was diagnosed in men and women each with a serum UA level over 7.0 mg/dL and 6.0 mg/dL [20].

### Statistical analysis

All statistical analyses were performed using the SAS ver. 9.3 (SAS Institute Inc., Cary, NC, USA). Significance testing of the difference between means was done by an independent Student’s *t*-test, and correlations were assessed by Pearson coefficients. Analysis of covariance was used to test for differences owing to covariates and multiple linear and logistic regression modeling to distinguish each of the contributions of age, diabetes, dyslipidemia, BMI, and eGFR to the effect of UA on BP and hyperuricemia on hypertension. All continuous variables were expressed as means ± standard deviation. A *p* value of <0.05 was considered statistically significant, but to compensate the potential of an alpha error generating from the stratified analysis regarding age and gender (a total of six groups) in the multiple linear and logistic regression model, Bonferroni correction was applied and a *p* value of 0.05/6 was considered statistically significant.

## Results

**Figure 2:**
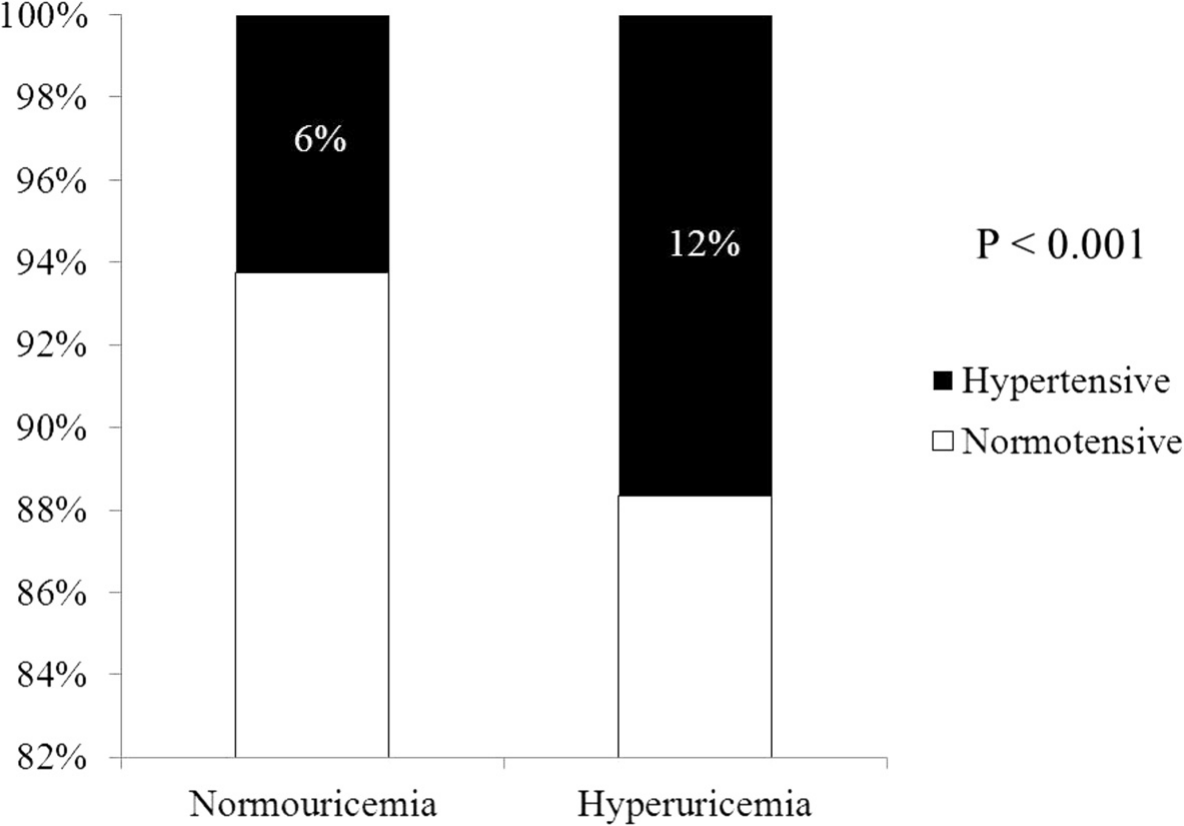
Prevalence of hypertension in hyperuricemia. A significant difference between normouricemic and hyperuricemic patients was seen among the total subjects who were enrolled (*p* < 0.05).

**Figure 3:**
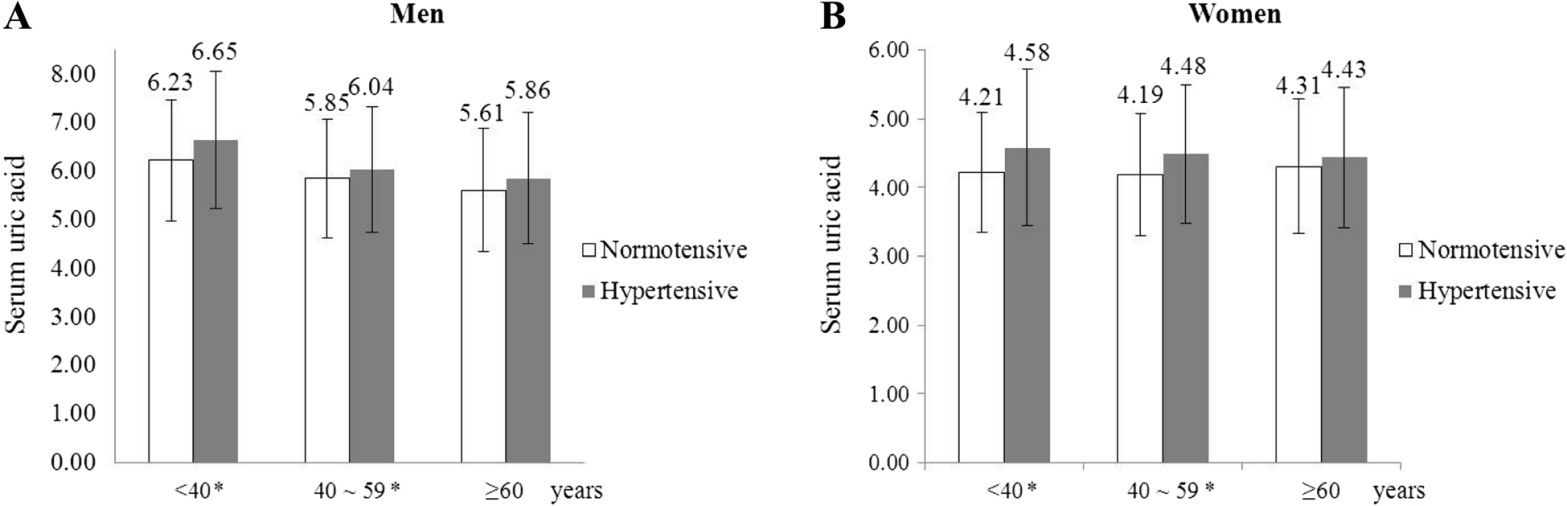
Serum uric acid levels in different age/gender groups. *Indicates groups with significant differences (*p* < 0.05) between normotensive and hypertensive patients. (**A**) Men. (**B**) Women.

A total of 45,098 subjects were included in the final analysis. Baseline characteristics of the subjects divided into three groups by age are presented in Table 1. Mean age and BMI of the total subjects were 39.0 ± 11.6 kg/m^2^ and 23.2 ± 3.4 kg/m^2^ each. Systolic BP was significantly higher in group ≥60 compared to the other groups (126.6 ± 15.3 mmHg vs. 114.7 ± 12.8 mmHg, 120.2 ± 14.2 mmHg, *p* < 0.001). Comparison of diastolic BP between the three groups also showed similar results (76.3 ± 9.7 mmHg vs. 70.8 ± 8.9 mmHg, 74.8 ± 9.9 mmHg; *p* < 0.001). The prevalence of comorbidities such as hypertension, diabetes, and dyslipidemia was highest in group ≥60, whereas the prevalence of hyperuricemia was highest in group <40. Nearly twice as many subjects were diagnosed with hypertension in hyperuricemic subjects than normouricemic subjects (12% vs. 6%, *p* < 0.001) (Figure 2). The comparison of serum UA levels of hypertensive subjects in different age/gender groups are presented in Figure 3. Serum UA levels were significantly higher in subjects with hypertension among all the age/gender groups except ≥60.

**Table 1:** **Baseline characteristics of each group by age**

**Variable**	**Total (** ***n*** **= 45,098)**	**<40 years (** ***n*** **= 28,864)**	**40–59 years (** ***n*** **= 13,118)**	**≥60 years (** ***n*** **= 3,116)**	***p*** **value**
Male	24,031 (53.3)	15,212 (52.7)	7,089 (54.0)	1,730 (55.5)	<0.001
Age (years)	39.0 ± 11.6	31.7 ± 4.9	48.7 ± 5.5	65.0 ± 4.2	<0.001
Height (cm)	165.6 ± 8.7	167.2 ± 8.5	163.5 ± 8.3	160.0 ± 8.6	<0.001
Weight (kg)	63.9 ± 12.6	64.3 ± 13.7	63.7 ± 10.2	60.5 ± 9.5	<0.001
Body mass index (kg/m^2^)	23.2 ± 3.4	22.9 ± 3.7	23.7 ± 2.8	23.6 ± 2.9	<0.001
Systolic blood pressure (mmHg)	117.1 ± 13.9	114.7 ± 12.8	120.2 ± 14.2	126.6 ± 15.3	<0.001
Diastolic blood pressure (mmHg)	72.3 ± 9.5	70.8 ± 8.9	74.8 ± 9.9	76.3 ± 9.7	<0.001
Uric acid (mg/dL)	5.2 ± 1.5	5.3 ± 1.5	5.1 ± 1.4*	5.1 ± 1.3*	<0.001
Fasting blood sugar (mg/dL)	91.3 ± 17.7	89.3 ± 14.6	94.4 ± 20.1	96.6 ± 21.4	<0.001
Estimated glomerular filtration rate (mL/min/1.73 m^2^)	88.0 ± 27.1	91.2 ± 28.0	83.1 ± 24.6	78.4 ± 23.4	<0.001
Total cholesterol (mg/dL)	180.2 ± 33.8	174.2 ± 31.3	189.9 ± 33.7	194.4 ± 34.4	<0.001
High-density lipoprotein (mg/dL)	52.0 ± 10.2	52.1 ± 10.0*	51.8 ± 10.4^†^	52.0 ± 10.4*^,†^	0.046
Triglyceride (mg/dL)	105.6 ± 82.5	100.1 ± 62.2	115.4 ± 67.0	115.3 ± 60.0	<0.001
Low density lipoprotein (mg/dL)	107.1 ± 29.3	102.3 ± 27.2	115.0 ± 30.7	117.3 ± 31.2	<0.001
Hypertension	3,122 (6.9)	1,144 (3.9)	1,377 (10.5)	601 (19.3)	<0.001
Diabetes mellitus	1,224 (2.7)	344 (1.2)	625 (4.8)	255 (8.2)	<0.001
Dyslipidemia	10,073 (22.3)	5,330 (18.5)	3,752 (28.6)	991 (31.8)	<0.001
Hyperuricemia	5,700 (12.6)	4,104 (14.2)	1,296 (9.9)	300 (9.6)	<0.001

Values are presented as number (%) or mean ± standard deviation.

All *p* values in the *post hoc* analysis comparing each group in all the variables were under 0.05 except the groups indicated as same letters like * or †.

According to the multivariate linear regression analysis between serum UA and BP, in men <40, serum UA was significantly associated with systolic (*β* = 0.25, *p* = 0.002) and diastolic BP (*β* = 0.41, *p* < 0.001) after adjustment for age, diabetes, dyslipidemia, BMI, and eGFR. However, men between the age 40 and 59 showed similar results regarding only diastolic BP (*β* = 0.43, *p* < 0.001). The association between serum UA and BP was stronger in women <40 (*β* = 0.54, *p* < 0.001 for systolic BP; *β* = 0.65, *p* < 0.001 for diastolic BP) and in between 40 and 59 regarding diastolic BP (*β* = 0.51, *p* < 0.001). The association was not significant in men and women ≥60 (Table 2).

**Table 2:** **Multivariate linear regression analysis between blood pressure and uric acid**

**Groups**	**Men**						**Women**					
	**SBP**			**DBP**			**SBP**			**DBP**		
	***β***	***R*** ^**2**^	***p*** **value**	***β***	***R*** ^**2**^	***p*** **value**	***β***	***R*** ^**2**^	***p*** **value**	***β***	***R*** ^**2**^	***p*** **value**
Total	0.33	0.10	<0.001	0.39	0.10	<0.001	0.61	0.19	<0.001	0.60	0.14	<0.001
<40 years	0.25	0.09	0.002	0.40	0.09	<0.001	0.54	0.05	<0.001	0.65	0.04	<0.001
40–59 years	0.29	0.06	0.03	0.43	0.05	<0.001	0.44	0.12	0.04	0.51	0.09	<0.001
≥60 years	0.57	0.05	0.05	0.34	0.03	0.08	−0.67	0.07	0.12	−0.26	0.04	0.35

Adjustment was done with age, diabetes, dyslipidemia, body mass index, and estimated glomerular filtration rate.

*SBP* systolic blood pressure, *DBP* diastolic blood pressure.

After performing multiple logistic regression analysis, the odds ratios (ORs) of hyperuricemia for hypertension were 1.25 (95% confidence interval [CI], 1.08 to 1.45; *p* = 0.003) and 1.33 (95% CI, 1.11 to 1.60; *p* = 0.002) each in men <40 and in 40 to 59, respectively. In women <40, the OR was 2.60 (95% CI, 1.37 to 4.94; *p* = 0.003). The relationship between hyperuricemia and hypertension was not significant in other age/gender groups (Table 3).

**Table 3:** **Multivariate logistic regression analysis between hyperuricemia and hypertension in different age/gender groups**

**Groups**	**Men**			**Women**		
	**OR**	**CI**	***p*** **value**	**OR**	**CI**	***p*** **value**
Total	1.29	1.16–1.44	<0.001	1.35	0.99–1.84	0.06
<40 years	1.25	1.08–1.45	0.003	2.60	1.37–4.94	0.003
40–59 years	1.33	1.11–1.60	0.002	1.26	0.81–1.95	0.31
≥60 years	1.29	0.93–1.79	0.13	1.17	0.65–2.09	0.61

Adjustment was done with age, diabetes, dyslipidemia, body mass index, and estimated glomerular filtration rate.

*OR* odds ratio, *CI* confidence interval.

## Discussion

The major findings in the present study enable us to identify the importance of age in the relation between serum UA and BP. Although serum UA and BP showed a significant relation in the overall population, when we evaluated it according to different age groups (<40, 40–59, ≥60), it was only significant in the non-elderly population under age 60 in both genders. There have been several studies suggesting that the strength of the relationship between serum UA and BP is more dominant in the younger age groups and decreases during the aging process as the duration of hypertension gets longer [1,6,12,21-23]. However, none of the previous studies have tried to investigate the effect of different age groups on the relation as a primary goal. To our knowledge, this is the first study to confirm the effect of different age groups on the relation between serum UA and BP in a single large cohort.

The prognostic significance of serum UA in different disease entities such as diabetes, chronic kidney disease, and cardiovascular diseases have been recognized in several previous studies [2,5,6,9,24,25]. Understanding the relationship between each diseases and serum UA has been important due to the potential benefit which could be achieved by applying it to new treatment strategies. Feig et al. [16] mentioned that early hypertension developed in children and adolescents with hyperuricemia could be reversed with urate reduction. Considering the practical implications in every day clinical practice, our results suggest the same could be applied beyond the adolescent to the non-elderly adults under age 60 with early hypertension and hyperuricemia.

There are several potential explanations for the attenuation of the relationship in the elderly. Previous studies implicating similar results have mentioned that the effect of a single estimate of serum UA in the young may decrease over time, and the higher background rate of hypertension incidence by other causes with increasing age may contribute to the reduction of the strength of the relationship between serum UA and BP [21]. Although most of the studies controlled possible confounding variables such as age, BMI, and serum cholesterol levels, many of them did not adjust for diabetes [6,25], antihypertensive therapy [24-26], or diuretic use [25-27] which led to controversial results regarding the relationship between serum UA and BP, especially in the elderly. However, our study initially was designed for complete control over the variables mentioned above and excluded patients taking medications possibly affecting serum UA levels and BP. Variables such as age, BMI, diabetes, dyslipidemia, and renal function, represented by eGFR, were all taken into account for adjustment in the final analysis. Even after the adjustments, the current study confirmed a significant correlation with serum UA and BP in the non-elderly patients under age 60. Hyperuricemia increased the risk of hypertension in both genders, but was only significant in men <60 and women <40 in the multiple logistic regression analysis. Particularly in women younger than 40 with hyperuricemia, our study demonstrated a 2.6-fold increase in the risk of hypertension. Stronger association of serum UA with BP and hypertension in women was seen, which is in line with previous studies [6,23,28,29]. Awareness and early interventional strategies in controlling hyperuricemia associated with BP reduction in this specific group may obtain maximal benefit.

Studies using animal models and cell cultures have revealed mechanisms by which UA could cause hypertension. In short, hypertension was developed by UA-mediated renal vasoconstriction resulting from a reduction in endothelial levels of nitric oxide, with activation of renin-angiotensin system [30,31]. Microvascular renal disease independently was caused by UA over time, inducing the development of hypertension [30].

Recent therapeutic trials with UA-lowering agents have demonstrated promising results for reducing BP. In one small study, the use of allopurinol, a xanthine oxidase (XO) inhibitor, resulted in reduction of BP in adolescents with newly diagnosed hypertension with hyperuricemia [16]. In another small study, UA-lowering therapy with either allopurinol or probenecid, a uricosuric agent, significantly reduced BP in prehypertensive, obese, adolescents with hyperuricemia irrespective of the UA-lowering mechanisms [32]. Allopurinol also showed significant additional BP reduction when it was combined with enalapril compared to enalapril alone in hyperuricemic hypertensive adolescents [33]. There was a report that a 6-month treatment of febuxostat, a more recently developed selective XO inhibitor, showed a significant BP reduction even in the elderly patients (mean age, 67 ± 10.3 years) with serum UA ≥ 8 mg/dL in contrast to allopurinol [34]. Febuxostat had a stronger UA-lowering effect and a renoprotective effect and was also superior to allopurinol at inhibiting oxidative stress atherogenesis, hypertension, and vascular endothelial damage. However, the study was performed with an intermediate size specific group who underwent cardiac surgery, and 90% of the patients were already being treated with antihypertensive therapy. Therefore, the effect of febuxostat on BP reduction may not be applicable to the general hyperuricemic population including the elderly, yet.

Our study has several limitations. First, its cross-sectional design could not reveal the causal role of serum UA in the development of hypertension. Increased UA levels at baseline are suggested to result in vascular dysfunction but after chronic exposure to high UA levels, the corresponding alterations in the vasculature diminish over time [35]. There may be an underlying genetic difference between genders but the results of this study demonstrating a stronger association between UA and hypertension in women than in men could have occurred due to the difference in the duration of exposure to elevated UA levels [36]. Second, in contrast with previous studies [37], the prevalence of hyperuricemia was highest in group <40. In previous studies, the increasing prevalence of hyperuricemia in the elderly was because of higher incidence of taking antihypertensive and/or diuretic agents [7,17]. The exclusion of these patients may have led to the results above. Third, the statistical insignificance in the elderly may have been due to the relatively smaller sample size compared to the other groups. Fourth, with the exclusion of subjects on antihypertensive medications, this may have generated a bias in which our study results only regard subjects with mild hypertension. However, our distinct study design compared to previous observational studies [24-27] in which we completely controlled medications affecting serum UA levels may provide a more accurate interpretation in the relationship between serum UA and BP. Finally, we cannot absolutely exclude the possibility that the study subjects were taking medications which could affect serum UA levels or BP from family or friends in secrecy because we only evaluated the medication history based on the legal prescriptions recorded by HIRA.

## Conclusions

This single large cohort study confirmed that the relationship between serum UA and BP, hyperuricemia and hypertension is quite different in each age group. In contrast to the elderly of 60 and over, the non-elderly showed a significant association between serum UA and BP in both genders. Hyperuricemia increased the relative risk of hypertension by approximately 30% in men under 60 and by 2.6 fold in women under 40. We believe the present study suggests specific age/gender groups which would obtain maximal benefit from the treatment of hyperuricemia for the prevention and treatment of hypertension.
